# An isogenic hiPSC-derived keratinocyte model reveals CXCL10/CXCL11 inflammatory dysregulation in epidermolysis bullosa simplex

**DOI:** 10.3389/fcell.2026.1918694

**Published:** 2026-08-21

**Authors:** Manoubia Saidani, Sabrina Martineau, Marwa Mahmoud, Sophie Domingues, Manon Doyen, Lina El Kassar, Smail Hadj-Rabia, Christine Bodemer, Jennifer Allouche, Christine Baldeschi, Nathalie Holic

**Affiliations:** 1 CECS/AFM, I- STEM, Corbeil-Essonnes, France; 2 INSERM/UEPS UMR 861, Paris Saclay University, I-STEM, Corbeil-Essonnes, France; 3 Human Medical Molecular Genetics Department, Human Genetics and Genome Research Institute, National Research Centre (NRC), Cairo, Egypt; 4 Stem Cell Research Unit, Medical Research Centre of Excellence, NRC, Cairo, Egypt; 5 Department of Dermatology, Reference Centre for Genodermatoses and Rare Skin Disease (MAGEC), Filière FIMARAD, Hopital Universitaire Necker-Enfants Malades, APHP-Centre, Paris, France; 6 Paris Cite University, Paris, France

**Keywords:** CXCL10/CXCL11, epidermolysis bullosa simplex, induced pluripotent stem cells, inflammation, keratinocytes

## Abstract

Epidermolysis bullosa simplex (EBS) is a genetic skin disorder driven by dominant pathogenic variants in *KRT5* or *KRT14* genes, leading to cytoskeletal fragility in basal keratinocytes and intraepidermal blistering. No curative therapies are currently available, and the link between keratin mutations and disease mechanisms remains incompletely understood. To further investigate the inflammatory component of EBS, we used a model of hiPSC-derived keratinocytes carrying dominant *KRT5* variants, alongside a genetically corrected isogenic counterpart. This approach established a direct link between the *KRT5* variants and keratin aggregation, impaired proliferation, and an inflammatory phenotype. The inflammatory signature was confirmed by increased expression of IL1A and IL1B, consistent with previous observations in EBS, while CXCL10 and CXCL11 emerged as newly identified dysregulated chemokines. Their consistent increase across independent cell lines, elevated secretion, and normalization in the CRISPR-corrected isogenic cells indicate that *KRT5* variants trigger a keratinocyte-intrinsic CXCL10/CXCL11 inflammatory response. Pharmacological inhibition of the IFN-γ-JAK1/2-STAT1 pathway suppressed their secretion, supporting JAK inhibition as a potential therapeutic strategy to modulate EBS-associated inflammatory dysregulation. In conclusion, this study shows that beyond structural defects, *KRT5* variants establish a keratinocyte-intrinsic inflammatory phenotype in which the CXCL10 and CXCL11 axis emerges as a key disease-associated signature and a promising therapeutic target.

## 1 Introduction

Epidermolysis bullosa simplex (EBS) is an inherited skin fragility disorder characterized by basal keratinocyte cytolysis, intraepidermal blistering, and epidermal mechanical vulnerability, most commonly caused by dominant *keratin 5* (*KRT5)* and *14* (*KRT14)* pathogenic variants (Mariath et al., 2020). Clinical severity varies widely among patients, influenced by genetic, epigenetic, and environmental factors (Annicchiarico et al., 2015; Bchetnia et al., 2024; Esposito et al., 2016). While EBS has long been attributed primarily to mechanical fragility of keratinocytes expressing mutant keratins, emerging evidence highlights an additional contribution of inflammatory mechanisms (Annicchiarico et al., 2015; Bchetnia et al., 2024; Esposito et al., 2016). Dysregulated inflammation appears to be an important component of disease pathophysiology (Bchetnia et al., 2024), with elevated levels of pro-inflammatory cytokines detected in serum and blister fluid in several epidermolysis bullosa (EB) subtypes (Annicchiarico et al., 2015; Esposito et al., 2016), suggesting that EB may involve a systemic inflammatory process rather than being limited to a strictly cutaneous mechanical disorder. This hypothesis is further supported by evidence that keratin intermediate filaments regulate cutaneous immunity, and that keratin dysfunction triggers immune dysregulation (Evtushenko et al., 2021; Hobbs et al., 2012). Anti-inflammatory therapies have yielded encouraging results in EBS (Wally et al., 2025). Topical diacerein (an orphan drug targeting IL1B-associated inflammatory signaling) achieved >40% blister reduction in 60% of 17 EBS patients (Wally et al., 2018), while Apremilast, (a phosphodiesterase-4 inhibitor with anti-inflammatory effects) reduced blisters in 2/3 generalized EBS cases (Castela et al., 2019). These clinical data support the relevance of inflammatory mechanisms in EBS and underscore unresolved aspects of keratinocyte-specific mechanisms, signaling cascades, and patient heterogeneity, all of which are likely to influence treatment efficacy.

Using a robust and well-established differentiation protocol, which enables the efficient generation of uniform basal keratinocyte populations from human induced pluripotent stem cells (hiPSC) (Domingues et al., 2022), we recently developed a cellular model based on hiPSC-derived keratinocytes from patients carrying dominant pathogenic *KRT5* variants (Coutier et al., 2022). This model faithfully recapitulates hallmarks of EBS, including proliferative defects and keratin filament aggregates. In the present study, building on this model, we investigated the inflammatory phenotype associated with dominant *KRT5* variants. The inclusion of a CRISPR-corrected isogenic line allowed us to directly assess whether the *KRT5* variants were causally linked not only to cytokine dysregulation but also to key pathological features, including impaired proliferation and keratin filament aggregation. Based on prior transcriptomic analyses, we first examined IL1A and IL1B as reference pro-inflammatory mediators previously implicated in EBS and related skin disorders. We then focused on CXCL10 and CXCL11, which emerged among the most strongly dysregulated genes and remain poorly characterized at the cellular level. Given that these chemokines are intrinsically dysregulated in *KRT5*-variant keratinocytes and are canonical targets of the IFN-γ-JAK1/JAK2-STAT1 signaling axis, we investigated whether this pathway contributes to CXCL10/CXCL11 dysregulation and evaluated whether its pharmacological inhibition could attenuate the inflammatory response. Collectively, these findings aim to deepen the understanding of the link between keratin defects and inflammatory phenotypes in EBS pathogenesis and to determine whether the CXCL10/CXCL11 axis may reveal therapeutically relevant targets.

## 2 Materials and methods

### 2.1 Source and culture of hiPSC

Patient-derived fibroblasts carrying *KRT5* mutations were purchased from Coriell, or EBS PBMCs/fibroblasts were obtained from the MAGEC center (Centre de référence des maladies rares de la peau et des muqueuses d’origine génétique) at Necker Hospital after informed consent (Declaration AC-2013-1973). The variant localization in the genome and the clinical phenotype were described in our previous study (Coutier et al., 2022). Cell lines were reprogrammed into hiPSC by Phenocell (Grasse, France) using OSKM episomal methods. HiPSC were maintained in StemPro® medium supplemented with stabilized FGF2 (10 ng/mL) on L7™ hPSC Matrix (10 μg/mL)-coated dishes, as previously described (Domingues et al., 2022).

### 2.2 Keratinocyte differentiation and culture

As previously described (Coutier et al., 2022), hiPSC clumps were seeded in hiPSC medium, then switched to dKSFM® (Invitrogen) supplemented with 0.5 nM BMP-4 and 1 μM retinoic acid on days 1 and 3. On day 5, medium was changed to dKSFM® alone until differentiation completion (days 20–30). Keratinocytes were enriched by differential trypsinization and expanded in CnT-NX-E™ medium (CELLnTEC) on collagen type I-coated flasks (15,000 cells/cm^2^) for up to two passages.

### 2.3 RT-qPCR (RT-quantitative PCR)

Total RNA was isolated using the RNeasy® Plus Mini Kit on QIAcube® (QIAGEN) per manufacturer’s protocol. RNA quantity and quality were assessed by NanoDrop. cDNA was synthesized from 500 ng RNA using SuperScript® III Reverse Transcription Kit (Invitrogen). RT-qPCR was performed on QuantStudio® using Luminaris® Color HiGreen qPCR Master Mix Low ROX (Invitrogen) with 25 ng cDNA per reaction and primers listed in Supplementary Table S3. Gene expression was quantified by the ΔΔCt method, normalized to 18S rRNA. Melting curve analysis confirmed product specificity and absence of non-specific amplification.

### 2.4 Western blotting

Cell pellets were lysed in RIPA lysis buffer (Sigma-Aldrich) supplemented with anti-proteases (Sigma-Aldrich) and anti-phosphatase (Roche). Protein concentrations were quantified by Pierce BCA protein assay (Thermo Scientific). Western blotting was performed using standard techniques with 4%–12% NuPAGE Novex gels (Invitrogen) and nitrocellulose membranes (Whatman, GE Healthcare). Blots were blocked in 5% BSA in PBS 0.1% Tween 20, incubated with anti-STAT1 (1:750; Cell Signaling) and anti-phospho-STAT1 Y701 (1:600; Cell Signaling), followed by fluorescent secondary antibodies (1:10,000; Supplementary Table S2). Quantitative normalization used anti-GAPDH (1:50,000; Cell Signaling). Proteins were imaged using Odyssey® CLx (LI-COR, United States) per manufacturer’s guidelines.

### 2.5 Osmotic shock

Keratinocytes were seeded at 15,000 cells/cm^2^ in 96-well plates. At 80% confluency, cells were treated with 150 mM urea for 5 min at 37 °C. Following medium change and 4 h recovery, cells were fixed and permeabilized in ice-cold methanol: acetone (1:1, v/v) and processed for immunostaining.

### 2.6 CRISPR/Cas9 keratin 5 rescue isogenic hiPSC line generation

Single-guide RNA (sgRNA) was designed using CRISPOR software (https://crispor.gi.ucsc.edu) and synthesized by IDT™ (Integrated DNA Technologies). Patient-derived hiPSC harboring the heterozygous c.536T>C *KRT5* variation (2.5 × 10^5^ cells) were electroporated with 150 pmol sgRNA (5′-TCA​TCG​ACA​AGG​TGA​GCT​AC-3′), 125 pmol *Streptococcus pyogenes* Cas9 (gift from Jean-Paul Concordet, MNHN/CNRS UMR 7196/INSERM U1154), 60 pmol single-stranded oligodeoxynucleotide (ssODN; IDT™) repair template, and 100 pmol Alt-R® Cas9 Electroporation Enhancer (IDT™) using the P3 Primary Cell 4D-Nucleofector® X Kit (Lonza) per manufacturer’s instructions. The ssODN contained 90 bp/40 bp left/right homology arms designed to revert the c.536T>C variation to wild-type sequence, with a silent nucleotide change in the protospacer adjacent motif (PAM) to prevent Cas9 re-cleavage post-correction. Electroporated hiPSC were plated in Matrigel® hESC-qualified Matrix-coated 24-well plates using StemMACS™ iPS-Brew XF medium + CloneR® (STEMCELL Technologies). At 48 h post-transfection, cells were dissociated and re-plated at low density for clonal expansion. After 10–15 days, individual hiPSC colonies were manually picked and screened for genome editing. Genomic DNA was extracted using QuickExtract™ DNA Extraction Solution (Lucigen) per manufacturer’s protocol. The target locus was amplified by PCR using Q5® High-Fidelity DNA Polymerase (New England Biolabs) with primers 5′-CAA​CCC​ACT​AGT​GCC​TGG​TT-3′ and 5′-CCG​TCC​CTC​TAA​AGC​TTT​TCT-3′ (98 °C 2 min; 35 cycles of 98 °C 10 s, 69 °C 20 s, 72 °C 2 min; 72 °C 2 min final extension). PCR products were verified by Sanger sequencing (Genewiz).

### 2.7 Pharmacological treatments

Interferon gamma (INF-γ) treatment: cells at 80% confluence were starved overnight and then treated with IFN-γ at 25 ng/mL (peprotech) for 8 h for transcriptomic analysis by collecting cell pellets and for 16 h for secreted proteins analysis by collecting supernatants.

Ruxolitinib treatment: cells at 80% confluence were starved overnight and then treated with ruxolitinib (0.8 μM, 2 h) prior to IFN-γ stimulation.

4-Phenylbutyrate (4-PBA) treatment: 10 days after keratinocyte differentiation induction, cell cultures were treated with 4-PBA (Sigma; 1 μM) every 2 days. During keratinocyte amplification, 4-PBA treatment was performed every 2 days until keratinocytes were confluent and then harvested for analysis. Supernatants were collected for ELISA analysis and pellets were performed for mRNA extraction and RT-qPCR analysis.

#### 2.8 Cytokine secretion analysis (ELISA)

Secreted mediators were detected in cell culture supernatants by ELISA. CXCL10 and CXCL11 were measured using Human CXCL10/IP-10, CXCL11 I-TAC, DuoSet ELISA reagents, (Bio-Techne R&D Systems) according to the manufacturer’s instructions.

## 3 Results

### 3.1 Transcriptional inflammatory dysregulation in EBS hiPSC-derived keratinocytes identifies CXCL10/CXCL11 as significantly altered mediators

**FIGURE 1:**
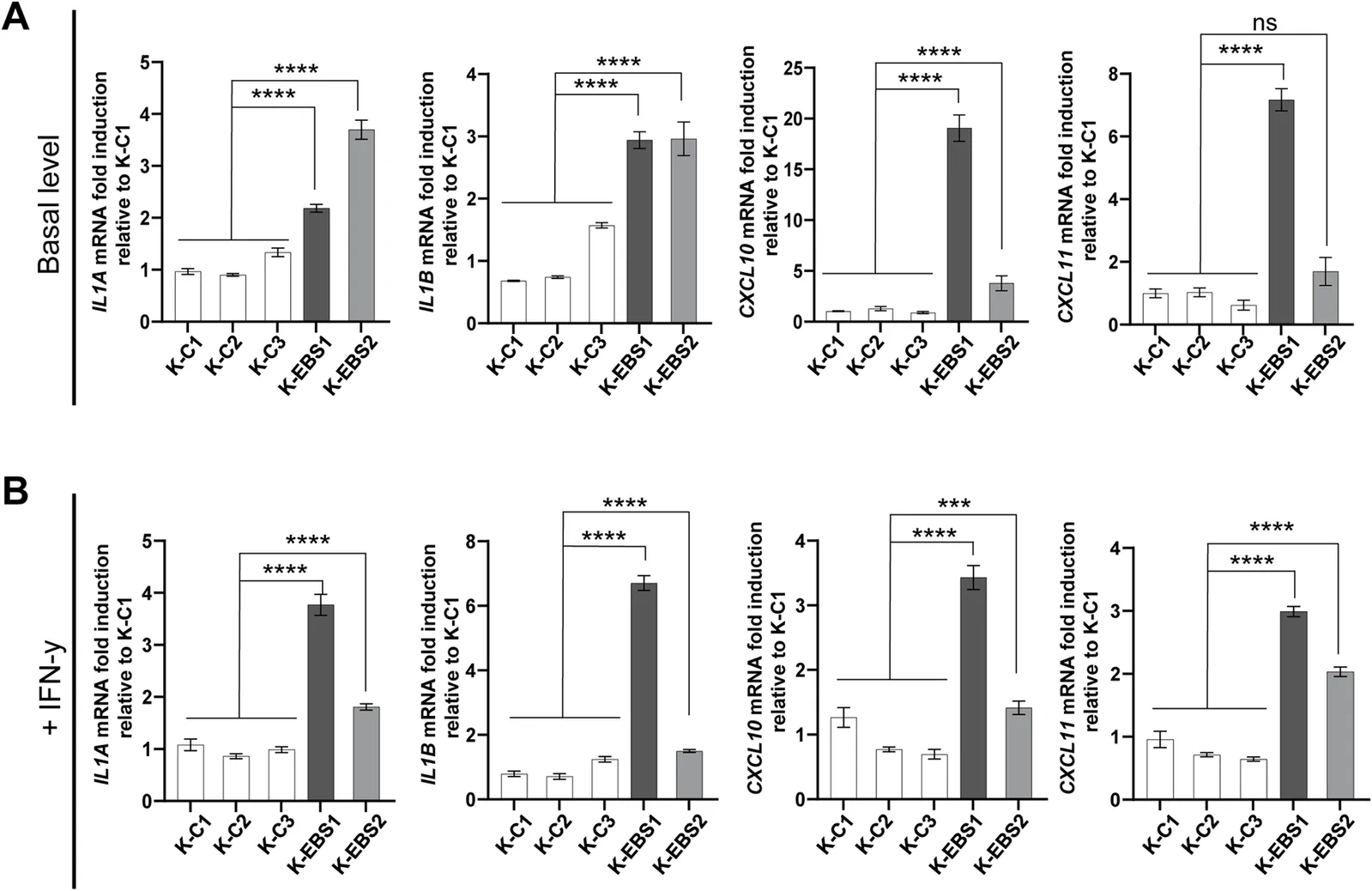
Inflammatory profile in EBS keratinocytes. RT-qPCR quantification of *IL1A, IL1B, CXCL10* and *CXCL11* expression under **(A)** basal level or **(B)** after treatment with IFN-γ (25 ng/mL, 8 h). Data are normalized to 18S and expressed as relative expression to control keratinocytes K-C1. Results are expressed as mean +/- SD (n = 3). Statistical analyses were performed using Mann-Whitney nonparametric t-test for side-by-side comparison. Each EBS hiPSC-derived keratinocytes (K-EBS) line was compared with the mean value of the control hiPSC-derived keratinocytes (K-C) lines. ns: non-significant, ***P < 0.005, and ****P < 0.001.

To further investigate the molecular mechanisms underlying *KRT5* variants in EBS, we previously performed a differential transcriptomic analysis of hiPSC-derived keratinocytes from EBS patients (K-EBS) and unaffected controls (K-C) revealing a significant enrichment in inflammation pathways (Coutier et al., 2022). To validate these findings, two K-EBS lines (K-EBS1, K-EBS2) and three control K-C lines (K-C1, K-C2, K-C3) were cultured under basal conditions or following IFN-γ stimulation. RT-qPCR analyses confirmed significant upregulation of *IL1A, IL1B, CXCL10* and *CXCL11* transcripts in K-EBS cells compared with controls in both conditions (Figures 1A,B). These results indicate that the inflammatory transcriptional signature is present in *KRT5*-variant keratinocytes under basal conditions and is further enhanced following IFN-γ stimulation. They also identify the CXCL10/CXCL11 axis as a consistent and previously underexplored inflammatory pathway in EBS.

### 3.2 Phenylbutyrate (PBA) partially modulates the inflammatory transcriptional profile of EBS keratinocytes, with a more consistent effect on CXCL10/CXCL11

**FIGURE 2:**
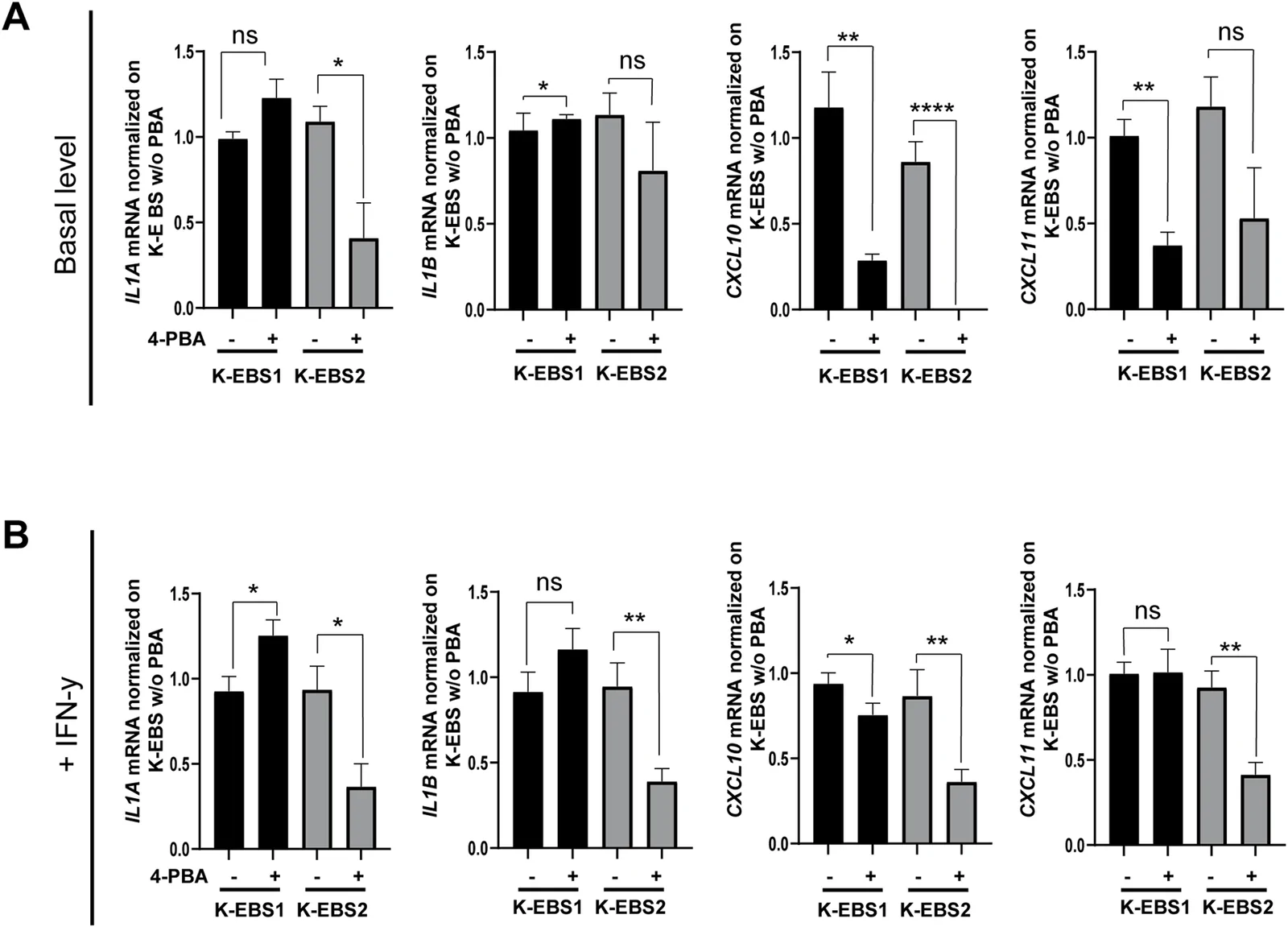
Modulation of inflammatory phenotype by 4-PBA in EBS keratinocytes. RT-qPCR quantification of *IL1A, IL1B, CXCL10,* and *CXCL11* expression in K-EBS1/K-EBS2 keratinocytes under basal conditions **(A)** or following IFN-γ stimulation **(B)** with (+) or without (−) 4-Phenylbutyrate (PBA). Expression levels were normalized to 18S rRNA and expressed as relative expression compared with the corresponding untreated K-EBS from the same K-EBS line. Data represent mean +/- SD (n = 3 biological replicates). Statistical analyses were performed using One sample t-test (hypothetical value = 1). *P < 0.05, **P < 0.01, ****P < 0.0001 and ns: non-significant.

To assess whether the transcriptional inflammatory phenotype observed in EBS keratinocytes could be pharmacologically modulated, we next evaluated the effect of 4-Phenylbutyrate (4-PBA), a chemical chaperone known to promote protein folding and reduce keratin aggregation in EBS keratinocytes (Chamcheu et al., 2011). We have previously showed that 4-PBA was able to rescue several pathological features in the hiPSC-derived EBS keratinocyte model (Coutier et al., 2022). We therefore investigated its impact on the inflammatory transcriptional profile of K-EBS1 and K-EBS2 cells (Figure 2). RT-qPCR analyses revealed a differential effect of 4-PBA on pro-inflammatory mediators. More specifically, 4-PBA significantly decreased *IL1A* and *IL1B* mRNA levels only in K-EBS2 cells under both basal and IFN-γ stimulated conditions whereas no significant modulation was observed in K-EBS1 (Figures 2A,B). In contrast, *CXCL10* and *CXCL11* transcripts were reduced by 4-PBA in both K-EBS lines under both conditions, indicating a more consistent effect on this chemokine axis (Figures 2A,B). Taken together, these results show that 4-PBA partially rescues the inflammatory transcriptional phenotype in EBS keratinocytes, with variable effects depending on the mediator and cell line. Importantly, whereas IL1A and IL1B responses were heterogenous, the consistent reduction of *CXCL10* and *CXCL11* transcripts in both EBS lines further supports the relevance of this pathway as a robust inflammatory readout in EBS keratinocytes.

### 3.3 Generation of an isogenic *KRT5*-corrected EBS hiPSC rescue line

**FIGURE 3:**
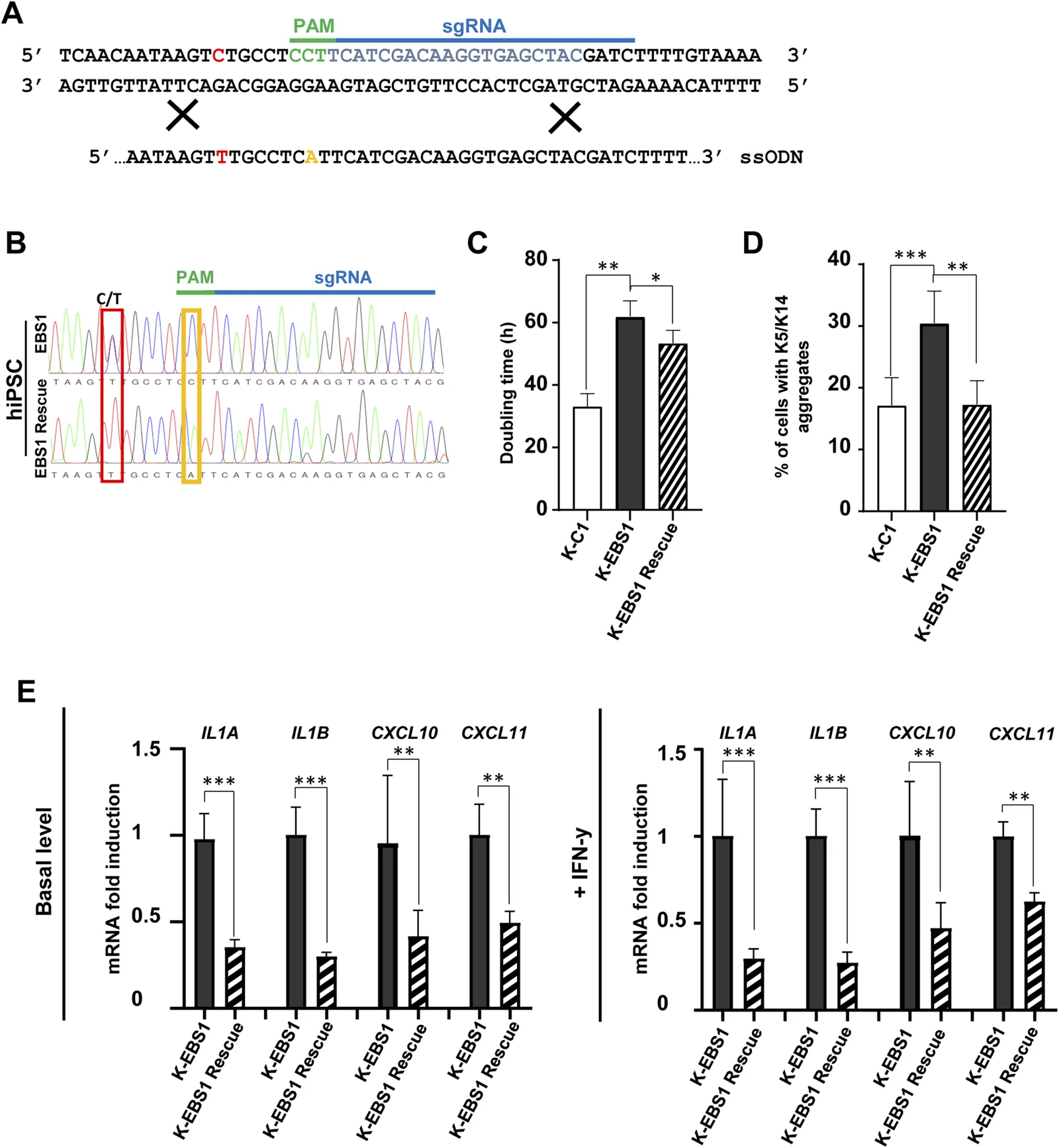
Correction of pathological structural phenotypes in KRT5-corrected keratinocytes. **(A)** Strategy for correcting the *KRT5* variant in EBS1. The c.536T>C variant, the single guide RNA (sgRNA) and the protospacer adjacent motif (PAM) are shown in red, blue and green, respectively. The single-stranded oligodeoxynucleotide (ssODN) carries the corrected “T” and a silent mutation (yellow). **(B)** Sequencing chromatograms of EBS1 and corrected clone (EBS1 rescue). **(C)** Expansion ratio (Cell number at day 9 relative to day 1; n = 2). **(D)** Percentage of keratinocytes showing at least one aggregate (n = 6–8, 20–50 cells/n). **(E)** RT-qPCR quantification of *IL1A, IL1B, CXCL10* and *CXCL11* expression in K-EBS1 and K-EBS1 rescue. (basal conditions: **l**eft panel, IFN-γ stimulation: right panel). Data are normalized to 18S and expressed as relative expression to K-EBS1. Histograms represent mean of data +/-SD, non-parametric Mann-Whitney test. *P < 0.05, **P < 0.01, ***P < 0.005.

While previous results indicate that the inflammatory transcriptional profile can be pharmacologically modulated, they do not establish whether this phenotype is directly driven by the *KRT5* variant. We therefore sought to determine the causal contribution of this variant to the inflammatory signature. To this end, we generated an isogenic hiPSC line by correcting the c.536T>C pathogenic variant in EBS1 hiPSC using CRISPR/Cas9-mediated homology-directed repair (Figure 3A). This approach enables precise genome-editing to produce isogenic cell lines differing only at the mutation of interest, thereby minimizing variability related to genetic background and other confounding factors. Briefly, a ribonucleoprotein complex composed of Cas9 nuclease and a single guide RNA targeting the c.536T>C variant was introduced into EBS1 hiPSC together with a single-stranded oligodeoxynucleotide donor containing the wild-type *KRT5* sequence flanked by homology arms (Figure 3A). The donor sequence also included a silent mutation within the protospacer adjacent motif to prevent re-cleavage after editing (Figure 3A). Following clonal selection, Sanger sequencing identified a corrected clone, designated EBS1-rescue, in which the wild-type sequence was successfully restored (Figure 3B). We were unable to generate an EBS2 rescue line due to sequence homology within the pathogenic variant-containing region with other keratin genes, which prevents design of a specific guide RNA.

The genomic integrity of the EBS1-rescue clone was confirmed by SNP genotyping (Supplementary Figure S1). Off-target (OFT) analysis of the ten most likely sites predicted by CRISPOR (Supplementary Table S1) revealed no detectable mutations, except for one located in the *KRT4* gene (OFT site 1, Supplementary Figure S2). As *KRT4* is not expressed in hiPSC or keratinocytes, this alteration is unlikely to impact downstream analyses. Similar to the parental EBS1 hiPSC line, the EBS1-rescue hiPSC line displayed typical pluripotent stem cell morphology with compact colonies and well-defined borders and expressed high levels (>90%) of pluripotency markers TRA-1–81 and SSEA-3 (Supplementary Figure S3). Altogether, these data confirm successful generation of a pluripotent isogenic EBS1-rescue line suitable for downstream keratinocyte differentiation and functional analyses.

### 3.4 Correction of *KRT5* leads to improvement of EBS-associated structural phenotypes

We next used this isogenic model to determine whether correction of the *KRT5* variant could restore structural phenotypes associated with EBS. For this purpose, the EBS1-rescue hiPSC were first differentiated into keratinocytes (K-EBS1-rescue) using a defined protocol. Briefly, hiPSC were seeded on matrix-coated plates in keratinocyte proliferation medium supplemented with retinoic acid and BMP4 on days 1 and 3, yielding a homogeneous population after ∼20 days and two passages. The K-EBS1-rescue keratinocytes displayed morphological and phenotypic characteristics comparable to those of the parental K-EBS1 line (Supplementary Figures S4A–C). They exhibited typical basal keratinocyte morphology characterized by packed cobblestone appearance (Supplementary Figure S4D). More than 95% of cells expressed Keratin 5 and Keratin 14 as assessed by flow cytometry (Supplementary Figure S4E) and further confirmed by immunostaining (Supplementary Figure S4F). Consequently, the K-EBS1 rescue line retains normal keratinocyte differentiation capacity.

We have previously established that K-EBS lines display reduced proliferation and stress-induced cytoplasmic keratin aggregates. Therefore, we evaluated the impact of *KRT5* gene correction on these two phenotypes. Cell expansion assays revealed a marked proliferation defect in K-EBS1 compared with controls, which was partially but significantly rescued in K-EBS1-rescue (Figure 3C). Furthermore, urea-induced osmotic stress followed by 4 h recovery showed a higher proportion of keratin aggregates in K-EBS1 than in controls, whereas K-EBS1-rescue cells exhibited aggregate levels comparable to controls (Figure 3D). These data demonstrate that CRISPR/Cas9-mediated correction of the *KRT5* gene reverses canonical EBS structural phenotypes, supporting a direct causal link between the *KRT5* pathogenic variant and the associated cellular phenotypes, such as proliferation and keratin aggregates.

### 3.5 Correction of *KRT5* normalizes inflammatory gene expression, with a marked rescue of the CXCL10/CXCL11 axis

We next used the isogenic model to determine whether correction of the *KRT5* variant could rescue the inflammatory signature observed in EBS keratinocytes. To assess this, K-EBS1 and K-EBS1-rescue keratinocytes were cultured under basal conditions or stimulated with IFN-γ, and *IL1A, IL1B, CXCL10,* and *CXCL11* mRNA levels were quantified by RT-qPCR. *KRT5* variant correction markedly reduced expression of all four mediators in K-EBS1-rescue compared with K-EBS1 under both conditions (Figure 3E). These findings indicate that the pathogenic *KRT5* variant directly contributes to inflammatory transcriptional dysregulation in EBS keratinocytes. Notably, because CXCL10 and CXCL11 were also consistently altered across independent EBS lines, these data further support the CXCL10/CXCL11 axis as a central component of the inflammatory phenotype.

### 3.6 Restoration of *KRT5* normalizes CXCL10 and CXCL11 secretion

**FIGURE 4:**
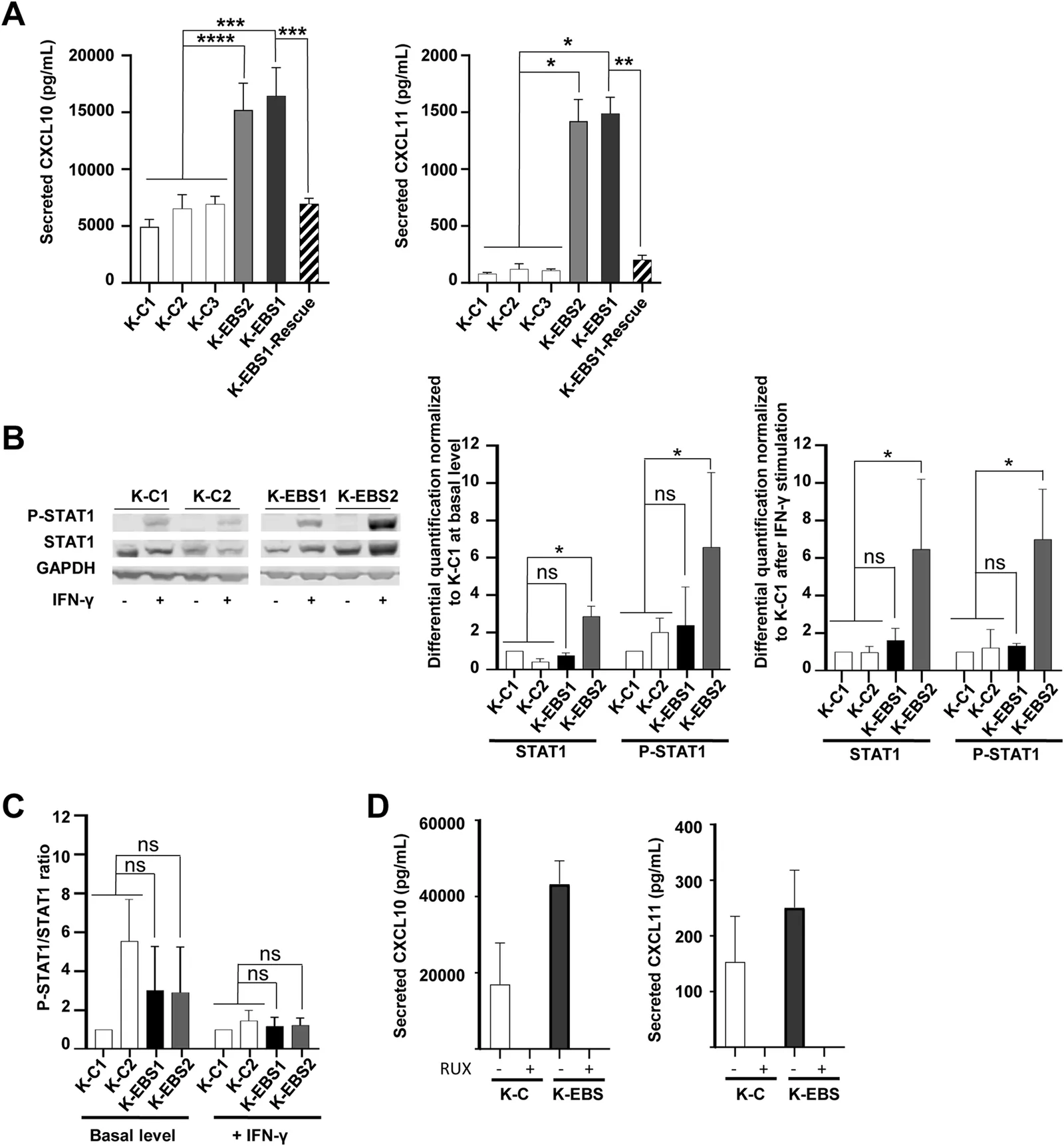
JAK/STAT1 pathway as therapeutic target in EBS Keratinocytes. **(A)** Enzyme-Linked Immunosorbent Assay (ELISA) quantification of secreted CXCL10 and CXCL11 in the supernatants from K-C, K-EBS Keratinocytes and K-EBS1-rescue treated with IFN-γ (25 ng/mL, 16 h). **(B)** Western blot of STAT1 and phospho-STAT1 (P-STAT1) in keratinocytes under basal or IFN-γ stimulated conditions (left: representative blot, right: densitometric quantification from 3 independent blots). STAT1 and P-STAT1 were normalized on GAPDH loading control and K-C1. **(C)** P-STAT1/STAT1 ratio in K-C and K-EBS1/2 normalized to K-C1. **(D)** ELISA of secreted CXCL10 and CXCL11 in IFN-γ stimulated keratinocytes supernatants +/- ruxolitinib (RUX). Bars represent mean of two K-C lines or K-EBS1/2 lines. Mann‒Whitney nonparametric t-test. *P < 0.05, **P < 0.01, ***P < 0.005, ****P < 0.0001, ns: non-significant.

To determine whether this intrinsic inflammatory phenotype was also reflected at the protein level, keratinocytes were cultured under IFNγ-stimulated conditions and cytokine secretion was quantified by ELISA. IL1A and IL1B secretion were increased in K-EBS2 compared with control keratinocytes, whereas no significant increase was detected in K-EBS1 under the experimental conditions tested (Supplementary Figure S5). We then assessed CXCL10 and CXCL11 secretion. Both K-EBS cell lines exhibited a marked increase in CXCL10 and CXCL11 secretion compared with K-C cells (Figure 4A), in agreement with the transcriptional upregulation previously observed. Importantly, correction of the *KRT5* variant in K-EBS1-rescue cells significantly reduced CXCL10 and CXCL11 levels in the supernatants of IFN-γ stimulated cells compared with parental K-EBS1 cells (Figure 4A), supporting a direct link between keratin dysfunction and CXCL chemokine overproduction. Together, these results suggest that the CXCL10/CXCL11 signature represents a reproductible hallmark of EBS keratinocytes.

### 3.7 JAK/STAT signaling contributes to CXCL10/CXCL11 secretion and represents a potential therapeutic target in EBS

The IFN-γ signaling pathway had already been associated with EBS through its link to keratin aggregate formation (Badowski et al., 2022). In addition, *CXCL10* and *CXCL11* represent canonical IFN-γ-stimulated genes induced via JAK1/2-mediated STAT1 phosphorylation (Supplementary Figure S6A). Given the consistent upregulation of CXCL10 and CXCL11, both at the transcriptional level and through increased secretion in EBS keratinocytes, we hypothesized that the IFN-γ signaling pathway might contribute to this inflammatory phenotype. Western blot analysis revealed elevated levels of total STAT1 and phosphorylated STAT1 (P-STAT1) in K-EBS2, whereas K-EBS1 levels remain comparable to K-C, under both basal and IFN-γ-stimulated conditions (Figure 4B). However, the ratio of phosphorylated STAT1 to total STAT1 (P-STAT1/STAT1) remains equivalent across K-EBS and K-C under all conditions (Figure 4C), suggesting that STAT1 activation is not consistently enhanced in EBS keratinocytes despite the differences in total STAT1 and P-STAT1 levels observed in K-EBS2.

To evaluate whether the IFN-γ pathway remains a potential therapeutic target, keratinocytes were pretreated prior to IFN-γ stimulation with ruxolitinib, a JAK1/2 inhibitor (Supplementary Figure S6A) already used to treat inflammatory dermatoses. Ruxolitinib abolished STAT1 phosphorylation in both K-C and K-EBS lines (Supplementary Figure S6B). ELISA analysis showed equivalent suppression of CXCL10 and CXCL11 secretion across all genotypes (Figure 4D), consistent with the complete inhibition of p-STAT1 by ruxolitinib. Together, these results indicate that, even in the absence of consistent STAT1 hyperactivation, CXCL10/CXCL11 production remains strongly dependent on JAK/STAT signaling, identifying this pathway as a therapeutically actionable node in EBS keratinocytes.

## 4 Discussion

While the genetic causes of EBS are well established, the mechanisms linking keratin pathogenic variants to downstream cellular alterations remain incompletely understood. EBS is primarily considered as a disorder of mechanical fragility resulting from abnormalities in the keratin cytoskeleton. However, beyond this primary structural defect, inflammatory alterations have also been reported in EBS and may represent an important disease-associated component (Bchetnia et al., 2024; Evtushenko et al., 2021).

In this study, we used hiPSC-derived keratinocytes from patients carrying dominant *KRT5* pathogenic variants to further investigate the inflammatory profile associated with EBS. In addition to canonical structural defects, including altered proliferation and keratin aggregation, *KRT5*-variant keratinocytes exhibited reproducible inflammatory alterations. Increased expression of *IL1A* and *IL1B* was observed, in line with previous reports in EBS patient samples and experimental models (Alexeev et al., 2017; Annicchiarico et al., 2015; Esposito et al., 2016; Lu et al., 2007; Wally et al., 2013). In addition to increased expression of *IL1A* and *IL1B*, increased secretion of both cytokines was detected in the culture supernatants of only one EBS keratinocyte cell line, indicating that *KRT5*-variant keratinocytes are capable of releasing these cytokines into the extracellular compartment. Given the intracellular and extracellular functions of IL1A and IL1B, our data suggest that extracellular IL1 signaling may contribute to the inflammatory phenotype of EBS keratinocytes. However, as intracellular IL1 levels and localization were not investigated, the relative contribution of intracellular versus extracellular IL1 signaling remains to be determined. Although these mediators were not the focus of the present study, their dysregulation confirms that our hiPSC-derived keratinocyte model reproduces key inflammatory features previously described in EBS. By contrast, CXCL10/CXCL11 emerged as the most robust and consistent alterations across the different experimental settings used. These chemokines were upregulated at the transcript level in independent EBS lines, increased at the protein level, reduced by 4-PBA, and normalized in the CRISPR-corrected isogenic line. This convergence of evidence identifies the CXCL10/CXCL11 axis as a central inflammatory signature in our model. Moreover, CXCL10 and CXCL11 were already upregulated under basal conditions, demonstrating that *KRT5* pathogenic variants establish a keratinocyte-intrinsic inflammatory phenotype. In this context, IFN-γ stimulation was used as an experimental inflammatory challenge to evaluate the amplification of this pre-existing inflammatory program rather than its initiating cause.

In line with these findings, previous studies have reported increased levels of cytokines and chemokines in EBS samples, including serum and blister fluid (Alexeev et al., 2017; Annicchiarico et al., 2015; Esposito et al., 2016) supporting the involvement of inflammatory pathways in disease progression. Among these, CXCL10 and CXCL11 have been reported only occasionally in serum of EBS patients and different types of EB and remain uncharacterized at the cellular level compared to IL1B pathways (Alexeev et al., 2017; Castela et al., 2019; Lettner et al., 2013). Our data extend these observations by showing that their dysregulation is present in a keratinocyte-only system and is normalized by genetic correction of the causal *KRT5* variant. This strongly supports the idea that CXCL10/CXCL11 upregulation is, at least in part, a keratinocyte-intrinsic consequence of keratin dysfunction rather than solely a secondary response to tissue damage.

The generation of a CRISPR-corrected isogenic hiPSC line represents a key strength of this study, as it minimizes genetic background effects and enables more confident attribution of phenotypic changes to the variant. Previous CRISPR/Cas9-mediated gene-editing approaches have demonstrated the feasibility of correcting variants in EBS models. Correction of EBS-causing variants has been demonstrated in *KRT14*-variant keratinocytes, resulting in reduced keratin aggregation (Kocher et al., 2017). More recently, correction of a *KLHL24* variant has been achieved in hiPSC; however, this work was limited to molecular characterization without phenotypic analysis (Ramovs et al., 2021). To our knowledge, no previous study has examined inflammatory dysregulation in an isogenic context. Here, correction of the *KRT5* variant restores proliferation and keratin organization. It also attenuates CXCL10/CXCL11 dysregulation, supporting a direct link between keratin dysfunction and the observed inflammatory alterations.

In our previous work, 4-PBA reduced keratin aggregation and partially rescued several pathological features in EBS keratinocytes (Coutier et al., 2022). In the present study, 4-PBA also decreased CXCL10 and CXCL11 transcript levels, although its effects remained variable depending on the mediator and conditions. Similar context-dependent effects of 4-PBA on pro-inflammatory mediators have been reported in keratinocytes (Spörrer et al., 2019). The variable modulation of IL1A/IL1B, contrasted with the consistent reduction of CXCL10/CXCL11, further supports the latter as the most robust readout in our model.

Given that CXCL10 and CXCL11 are canonical interferon-γ-stimulated genes, we investigated the involvement of the IFN-γ-JAK1/2-STAT1 signaling pathway. This pathway has previously been implicated in EBS models, particularly in relation to keratin aggregation (Badowski et al., 2022). The ratio of phosphorylated to total STAT1 remained unchanged, suggesting that STAT1 activation is not significantly increased in KRT5-variant keratinocytes. Therefore, the increased secretion of CXCL10/CXCL11 cannot be explained solely by enhanced STAT1 activation and likely involves additional regulatory mechanisms. Nevertheless, pharmacological inhibition of JAK1/2 with ruxolitinib completely suppressed CXCL10 and CXCL11 secretion, indicating that this pathway remains functionally required for their production and can be efficiently targeted.

In EB, particularly in inflammatory forms such as dystrophic EB pruriginosa, the JAK/STAT pathway has already emerged as a potential therapeutic target, with preliminary evidence from limited clinical reports indicating that JAK inhibitors can significantly improve pruritus and inflammatory skin lesions in refractory patients (Jiang et al., 2021; Chen et al., 2022). The increased secretion of CXCL10 and CXCL11 highlights their important role not only in EB but also in other inflammatory skin diseases. For example, in atopic dermatitis and psoriasis, treatments modulating the JAK/STAT pathway have emerged as major therapeutic options, as many key cytokines act through this pathway, and JAK inhibition provides both rapid antipruritic and anti-inflammatory effects (O’Shea et al., 2015; Krueger et al., 2016; Cui et al., 2026). In this broader context, the CXCL10/CXCL11 axis identified in EBS may reflect a shared inflammatory response module that can be pharmacologically controlled, despite a distinct primary trigger. Importantly, these findings do not challenge the primary mechanical nature of EBS but instead support the contribution of inflammation as a potentially targetable component of the disease. Accordingly, targeting downstream inflammatory pathways such as JAK/STAT may represent a complementary strategy to mitigate disease features not addressed by approaches aimed at restoring keratin integrity.

A limitation of the current study is the absence of immune cells, which play a key role in EBS pathophysiology, particularly inflammation and tissue repair (Bchetnia et al., 2024). Further studies incorporating immune components and more complex or *in vivo* systems will therefore be required to validate and extend our findings. This study highlights potential mechanisms driving CXCL10 and CXCL11 dysregulation in EBS keratinocytes. Keratin aggregation induces cellular stress (Yoneda et al., 2004; Chamcheu et al., 2011), which can activate inflammatory pathways such as NF-kB signaling. In addition, unfolded protein response pathways have been shown to promote cytokine production in epithelial cells (Grootjans et al., 2016; Smith, 2018; Gendrisch et al., 2022). Furthermore, STAT1-independent regulation of CXCL10 has also been described via NF-kB dependent mechanisms (Yeruva et al., 2008; Schroeder et al., 2017). Together, these mechanisms may cooperate to amplify upregulation in *KRT5*-variant keratinocytes, although further studies are needed to clarify their respective contributions.

In conclusion, this study shows that, beyond structural fragility, dominant *KRT5* variants induce a reproducible inflammatory program in hiPSC-derived keratinocytes. Within this program, the CXCL10/CXCL11 chemokines emerge as the most robust and variant-dependent inflammatory signature. The significant normalization following CRISPR correction and suppression by ruxolitinib positions them as a mechanistically relevant readout and a potential therapeutic target in EBS.

## Data Availability

The raw data supporting the conclusions of this article will be made available by the authors, without undue reservation.
