# Supplementary material for: An isogenic hiPSC-derived keratinocyte model reveals CXCL10/CXCL11 inflammatory dysregulation in epidermolysis bullosa simplex
Source: Front Cell Dev Biol. 2026 Aug 21;14:1918694. doi: 10.3389/fcell.2026.1918694 (PMC13541660; doi:10.3389/fcell.2026.1918694)
Supplement: Supplementary file 1 [file DataSheet1.docx]

Supplementary Material

**An isogenic hiPSC-derived keratinocyte model reveals CXCL10/CXCL11 inflammatory dysregulation in epidermolysis bullosa simplex**

Manoubia Saidani^1,^**^✝^**, Sabrina Martineau^2,^**^✝^**, Marwa Mahmoud^3,4^, Sophie Domingues^1^, Manon Doyen^2^, Lina El Kassar^1^, Smail Hadj-Rabia^5,6^, Christine Bodemer^5,6^, Jennifer Allouche^2^, Christine Baldeschi^2,^**^✝✝^**^,*^ , Nathalie Holic^2,^**^✝✝^**^,*^

^1^CECS/AFM, I- STEM, Corbeil-Essonnes, France

^2^INSERM/UEPS UMR 861, Paris Saclay University, I-STEM, Corbeil-Essonnes, France

^3^Human Medical Molecular Genetics Department, Human Genetics and Genome Research Institute, National Research Centre (NRC), Cairo, 12622, Egypt.

^4^Stem Cell Research Unit, Medical Research Centre of Excellence, NRC, Cairo, Egypt.

^5^Department of Dermatology, Reference Centre for Genodermatoses and Rare Skin Disease (MAGEC), Filière FIMARAD, Hopital Universitaire Necker-Enfants Malades, APHP-Centre, Paris, France.

^6^ Paris Cite University, Paris, France.

**^✝^** These authors contributed equally to this work and share first authorship

**^✝✝^** These authors contributed equally to this work and share last authorship

*** Correspondance** [cbaldeschi@istem.fr](mailto:cbaldeschi@istem.fr); [nholic@istem.fr](mailto:nholic@istem.fr)

**1. Supplementary material and methods**

**Off-target analysis**

We used the online tool CRISPOR (<https://crispor.tefor.net/>) to identify the ten most-likely off-target regions that could be targeted by the sgRNA (Supplementary Table S1). The region surrounding each off-target site was amplified by PCR using specific primers listed in Supplementary Table S4. PCR reactions were performed using the Q5 polymerase high fidelity (New England Biolabs) with 10 ng genomic DNA, 1 cycle of 2 min at 98°C, 30 cycles of 10 sec at 98 °C, 20 sec at 60-66 °C and 2 min at 72 °C, with a final 2 min extension at 72 °C. PCR products were then sequenced by Sanger sequencing (Genwiz) and compared to the unedited hiPSCs.

**SNP genotyping**

High quality genomic DNA was obtained from 5.10^6^ cells using QIAcube™ workstation using QIAamp® DNA Blood Mini Kit DNA (Qiagen). Genomic DNA hybridation was achieved on Infinium Core-24v1-2 BeadChip (Illumina). Data were analyzed with GenomeStudio v2.0.5 software (Illumina).

**Immunofluorescence**

Keratinocytes were fixed in 4% paraformaldehyde (Euromedex), permeabilized with 0.1% Triton X-100 (Sigma-Aldrich), and blocked with 5% BSA (Sigma-Aldrich). Samples were incubated with primary antibodies, overnight at 4°C, washed, then incubated 1 h with species-specific fluorophore-conjugated secondary antibodies and DAPI (1 μg/mL; Millipore) for nuclear counterstaining (antibodies: Supplemental Table S2). Images were acquired with a confocal microscope (LSM880-Airyscan, Carl Zeiss, Oberkochen, Germany).

**Flow Cytometry**

Experiments were performed using a MACSQuant® system (Miltenyi Biotec) with data analyzed in FlowJo software (15,000 events/sample). Acquisition parameters were optimized using a control cell line and maintained consistently across all samples. Keratinocytes were harvested with 0.05% Trypsin-EDTA, fixed for 15 min at room temperature in FCM Fixation Buffer (Santa Cruz Biotechnology), and stained. HiPSCs were dissociated with Accutase® and analyzed directly. All cell types were incubated for 1 h in PBS + 2% FBS with fluorochrome-conjugated antibodies (Supplemental Table S2), washed, and processed for analysis.

**Cytokine secretion analysis (ELISA)**

To promote IL1B secretion, keratinocytes were cultured to approximately 80% confluence, serum-starved overnight, and stimulated with IFN-γ (25ng/ml) for 4 h, followed by treatment with 20 µM nigericin (Sigma) for an additional 4 h. Cell culture supernatants were then collected for cytokine analysis. Secreted IL1A and IL1B were quantified by ELISA using the Human DuoSet ELISA kits for IL1A and IL1B (R&D Systems, Bio-Techne), according to the manufacturer's instructions.

**Supplementary Figures and Tables**

**2.1	Supplementary Figures**

**Supplementary Figure S1**

**Supplementary Figure S1: Genomic SNP array analysis showing EBS1 rescue hiPSCs**

**Supplementary Figure S2**

**Supplementary figure S2: Off-target analysis of EBS1 rescue hiPSC by Sanger sequencing**.

Sequence alignment of the ten most likely off-target (OFT) sites **(A)** K4, **(B)** K1, **(C)** CNTN4, **(D)** K77, **(E)** K79, **(F)** K6C, K6B and K6A, **(G)** CTNND2 and **(H)** K8P4) for the parental EBS1 and EBS1 rescue hiPSCs. OFT sites are underlined. The potential off-target sites 6 to 8 correspond to the same sequence but located in 3 different regions of the genome. Predicted OFT sites and primers used are listed in Supplementary Table S1 and Supplementary Table S4.

**Supplementary Figure S3**

**Supplementary figure S3 : characterization of EBS1 and EBS1 rescue isogenic hiPSC**

Representative phase-contrast of EBS1 (A) and EBS1 rescue isogenic (C) hiPSC (bar 50µm). Flow cytometry analysis of expression of TRA1-81 and SSEA-3 pluripotency markers in EBS1 (B) and EBS1 rescue isogenic (D) hiPSC.

**Supplementary Figure S4**

**Supplementary figure S4 : characterization of EBS1 and rescue EBS1 keratinocytes**

Representative phase-contrast image for K-EBS1 (A) and K-EBS1 rescue (D) (bar 20µm). Flow cytometry analysis of K5 and K14 expression in (B) K-EBS1 rescue (E). Representative immunostaining images (bar 20µm) for K5 (green) and K14 (red) of K-EBS1 (C) and K-EBS1 rescue (F) .

**Supplementary Figure S5**

**Supplementary figure S5 : IL1A and IL1B secretion**

ELISA quantification of secreted IL1A and IL1B in the supernatants from K-C and K-EBS Keratinocytes treated with IFN-γ. Results are expressed as mean ± SD (n = 3). Statistical analyses were performed using Mann‒Whitney nonparametric t-test for side-by-side comparison. Each K-EBS line was compared to K-C mean. ***P < 0.005, and ****P < 0.001, ns: non-significant.

**Supplementary Figure S6**

**Supplementary figure S6 : Ruxolutinib (RUX) inhibition**

**(A)** Schematic representation of IFN-γ signaling pathway showing ruxolitinib inhibition of JAK1/2. (Adapted from Ghosh and al Developmental cell 2024). **(B)** Western blot of p-STAT1 and STAT1 (GAPDH loading control) in IFN-γ-stimulated keratinocytes ± Ruxolitinib pretreatment.

**2.2 Supplementary Tables**

**Supplementary Table S1: List of the top 10 potential off-target sites for the sgRNA.**

The potential off-target sites 6 to 8 correspond to the same sequence but located in 3 different regions of the genome. The mismatched bases in putative off-target sites are indicated in red. Off-target sites are sorted by the CFD score.

| **sgRNA** | **Position** | **Off-target sequence** | CFD | **Gene** |
| --- | --- | --- | --- | --- |
| **Site 1** | chr12:53207372-53207394 | ACAGCTCACCTTGTCGATGA | 0.77 | *KRT4* |
| **Site 2** | chr12:53073533-53073555 | GAAACTCACCTTGTCAATGA | 0.76 | *KRT1* |
| **Site 3** | chr3:2637231-2637253 | GTAGCCTACCTTGTCAATGA | 0.74 | *CNTN4* |
| **Site 4** | chr12:53096667-53096689 | GGTGCTCACCTTGTCAATGA | 0.59 | *KRT77* |
| **Site 5** | chr12:53227559-53227581 | TTGGCCCACCTTGTCGATGA | 0.55 | *KRT79* |
| **Site 6** | chr12:52866973-52866995 | CTGGCTCACCTTGTCGATGA | 0.44 | *KRT6C* |
| **Site 7** | chr12:52845314:52845336 | CTGGCTCACCTTGTCGATGA | 0.44 | *KRT6B* |
| **Site 8** | chr12:52886424-52886446 | CTGGCTCACCTTGTCGATGA | 0.44 | *KRT6A* |
| **Site 9** | chr5:11892268-11892290 | GCAGCTTAGCTTGTCGATGA | 0.43 | *CTNND2* |
| **Site 10** | chr8:90426957-90426979 | GCAGTATACCTTGTCGATGA | 0.38 | *KRT8/P4* |

**Supplementary Table S2: Antibodies used for flow cytometry, immunocytochemistry and western-blotting**

**Supplementary Table S3: Primers used for RT-qPCR**

| **Gene** | **Forward (5’- 3’)** | **Reverse (5’- 3’)** |
| --- | --- | --- |
| **IL1A** | TCACGGCTGCTGCATTACAT | GGTCTTCATCTTGGGCAGTCA |
| **IL1B** | TGAGCTCGCCAGTGAAATGA | GGTCGGAGATTCGTAGCTGG |
| **CXCL10** | AGGAACCTCCAGTCTCAGCA | TGATGCAGGTACAGCGTACAG |
| **CXCL11** | CCTGGGGTAAAAGCAGTGAA | TGGGATTTAGGCATCGTTGT |
| **18S** | GAGGATGAGGTGGAACGTGT | TCTTCAGTCGCTCCAGGTCT |

**Supplementary Table S4: Primers used for off-target analysis**

| **sgRNA** | **Forward (5’- 3’)** | **Reverse (5' - 3’)** |
| --- | --- | --- |
| **Site 1** | ACCCCAGGACTCAGGACCCC | CAGCTCGCTCTCTGCAGCCA |
| **Site 2** | CGGCAGCCCTACCAGTGCAA | CGCAGTGGAGGAGGTGGTGG |
| **Site 3** | TGACCAGCCTTGAACCAGGTGA | AGCGCCTGAGGGTCTCTGGG |
| **Site 4** | GCACGATGTGGTGAGAGGTGGT | GCAGGCTCTGGTGGTGGGAG |
| **Site 5** | CCACGTCTCGGCATCCCAGG | AGCTCCAACTCTGCCAGCGG |
| **Site 6** | TGCCCATCTGTGCTGCCTCC | GCTTTGGCAGCCGCAGTCTG |
| **Site 7** | TGCCCATCTGTGCTGCCTCC | GCTTTGGCAGCCGCAGTCTG |
| **Site 8** | TGCCCATCTGTGCTGCCTCC | GCTTTGGCAGCCGCAGTCTG |
| **Site 9** | TGCACATCACAAAACCTGACACCT | GCTCTGGCCCAAAGAAGGTGC |
| **Site 10** | AGCCAGCATCTGCAGCTCCT | ACTGGCATGGGTCTGGGTGG |
